# Surgical Treatment of Peri-Implantitis: A 17-Year Follow-Up Clinical Case Report

**DOI:** 10.1155/2015/574676

**Published:** 2015-05-12

**Authors:** Fabrizio Bassi, Pier Paolo Poli, Davide Rancitelli, Fabrizio Signorino, Carlo Maiorana

**Affiliations:** Department of Dental Implants, Maxillofacial Surgery and Odontostomatology Unit, Fondazione IRCCS Cà Granda, University of Milan, Via della Commenda 10, 20122 Milan, Italy

## Abstract

The purpose of the present case report was to describe the surgical treatment of a peri-implantitis lesion associated with a regenerative approach. A 48-year-old patient came to authors' attention 36 months after the placement of a dental implant (ITI-Bonefit Straumann, Waldenburg, Switzerland) in position 46. A swelling of the peri-implant soft tissues was observed, associated with bleeding on probing and probing depth > 10 mm. A significant peri-implant bone loss was clearly visible on the periapical radiograph. A nonsurgical periodontal supportive therapy was firstly conducted to reduce the inflammation, followed by the surgical treatment of the defect. After mechanical and chemical decontamination with tetracycline solution, a regenerative approach consisting in the application of deproteinized bovine bone mineral (Bio-Oss, Geistlich Pharma AG, Wolhusen, Switzerland) and a collagen membrane (Bio-Gide, Geistlich Pharma AG, Wolhusen, Switzerland) was performed. An antibiotic therapy was associated with the treatment. The 17-year follow-up showed a physiological probing depth with no clinical signs of peri-implant inflammation and bleeding on probing. No further radiographic bone loss was observed. The treatment described in the present case report seemed to show improved clinical results up to a relevant follow-up period.

## 1. Introduction

While peri-implant mucositis describes a reversible inflammatory lesion limited to the mucosa, peri-implantitis also affects the supporting bone circumferentially around an osseointegrated dental implant [1]. However, although these definitions are universally accepted, the diagnostic criteria still raise doubts. The critical parameter in the diagnosis of peri-implant mucositis is bleeding on gentle probing (<0.25 N). Peri-implantitis lesions are characterized by irreversible changes in the crestal bone levels in conjunction with bleeding on probing (BOP) with or without concomitant worsening of peri-implant pockets. Furthermore, suppuration is a common finding in peri-implant affected sites [2].

Peri-implant disease recognises a bacterial etiopathogenesis. The microbiota associated with peri-implant soft tissues has been identified in many cross-sectional studies. Particularly, it has been proved that gram-positive facultative cocci and rods represent the dominating bacterial composition [3–5]. However, gram-negative anaerobic rods may also be found in small numbers and in low proportions. An endoscopic clinical study by Wilson Jr. demonstrated that even the presence of submucosal dental cement could have a positive relationship with peri-implantitis, as the foreign body gives rise to a bacterial infection [6]. With respect to the etiopathogenesis of implant failures, also nonmicrobial events have to be taken into account, including implant fractures as a consequence of occlusal overloading [7, 8].

As in cases of periodontitis, peri-implant infections may take years to develop. It could be assumed that the susceptibility to periodontal disease may be translated into predisposition to peri-implantitis. Accordingly, longitudinal studies have investigated the transmission of putative periodontal pathogens from periodontal to implant sites [3, 9, 10]. These findings stress the importance of eliminating potential reservoirs of periodontal pathogens before implant placement maintaining at the same time a periodontal health status in partially dentate patients with oral implants.

Several detoxification procedures have been proposed to decontaminate peri-implantitis affected implant surfaces, including air-powder abrasion, saline wash, citric-acid application, laser therapy, peroxide treatment, ultrasonic/manual debridement, and application of topical medication; however a definite gold standard could not be identified [11].

In case of a surgical approach, the primary purpose is to obtain access for debridement and decontamination of the infected implant surface. Furthermore, the apposition of bone substitutes might be recommended when the type of bone defect is suitable for a regenerative procedure; a well-defined crater-like defect may improve retention of the bone graft thereby allowing for an optimal healing [12]. Regenerative surgical therapy associated with the use of autogenous bone graft has demonstrated positive results up to 3 years [13]. Studies employing combinations of bone grafts, bone substitutes, and membranes have reported clinical and radiographic improvements over 3 to 4 years [14–16].

The purpose of the present case report was to show the long-term outcome of a surgical peri-implantitis treatment associated with regenerative procedures, focusing on the importance of both implant surface decontamination by means of tetracycline applications and reconstructive therapy with particulate heterologous bone graft.

## 2. Case Report

**Figure 1:**
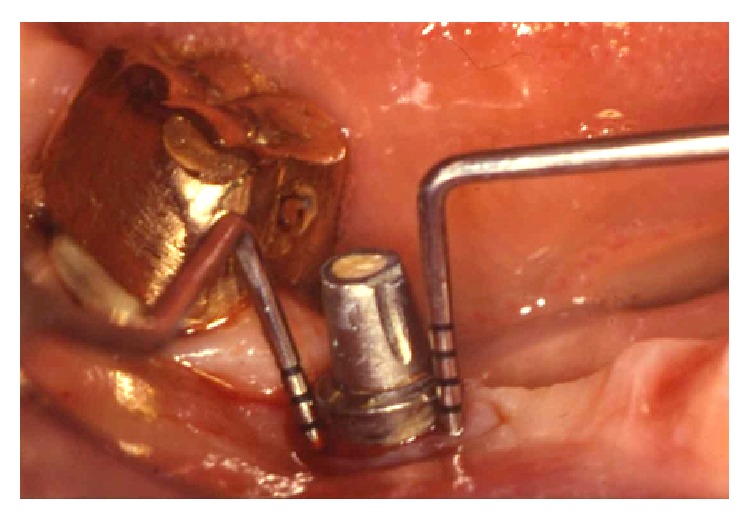
Clinical situation of the peri-implantitis affected implant, before treatment procedures. A mesial PD of 11 mm and a distal PD of 12 mm were recorded.

**Figure 2:**
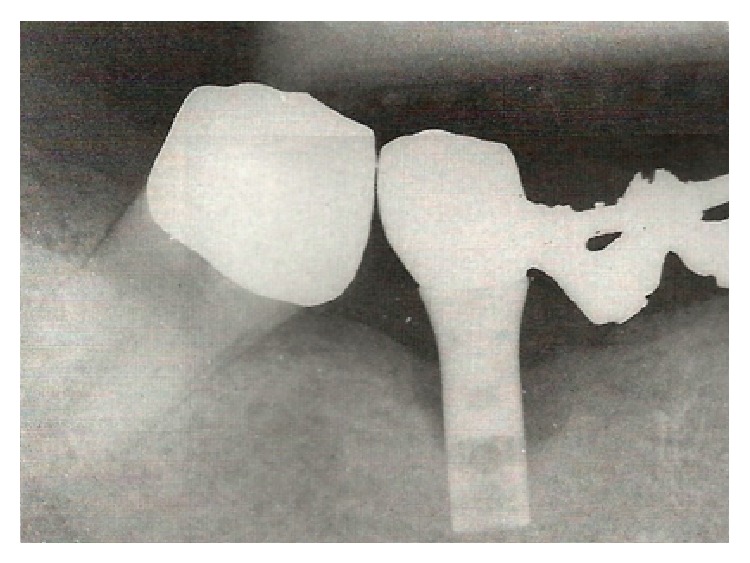
Preoperative intraoral radiography. A typical peri-implantitis crater-like defect was evident.

A 48-year-old female patient in good general health, with no local or systemic contraindications to oral surgery, without known allergies or sensitivities to medications, came to authors' observation in 1997. The patient showed swelling of the peri-implant soft tissues, associated with BOP and probing depth (PD) > 10 mm around an ITI-Bonefit (Straumann, Waldenburg, Switzerland) implant (4.1 mm in diameter and 12 mm in length), placed three years earlier in position 46 (Figure 1). The defect consisted in a circumferential bone resorption associated with a buccal dehiscence under maintenance of the lingual cortical plate, according to Class Ic (Schwarz et al. defect classification [12]). No clinical signs of mobility were recorded; however the periapical radiograph revealed a radiolucent area delimitating the implant (Figure 2).

**Figure 3:**
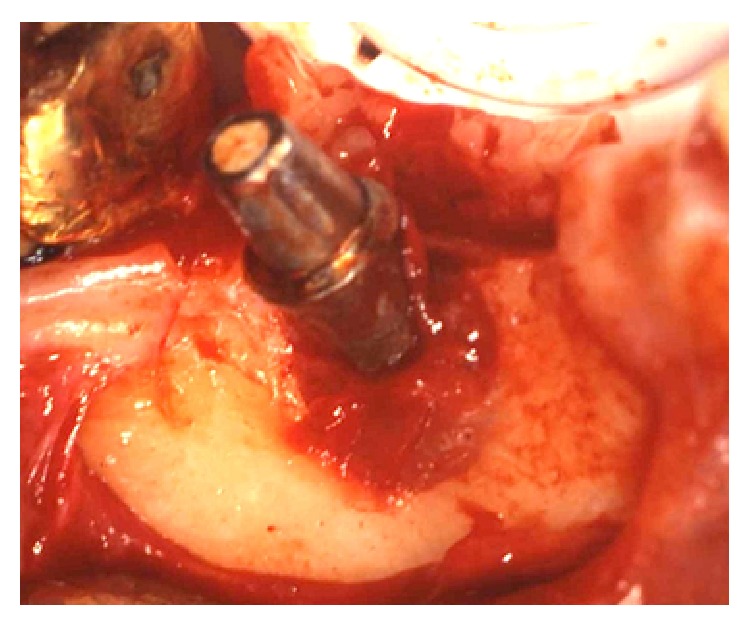
Clinical situation after the elevation of a mucoperiosteal flap. Granulation tissue delimitating the peri-implantitis defect was clearly visible around the implant.

**Figure 4:**
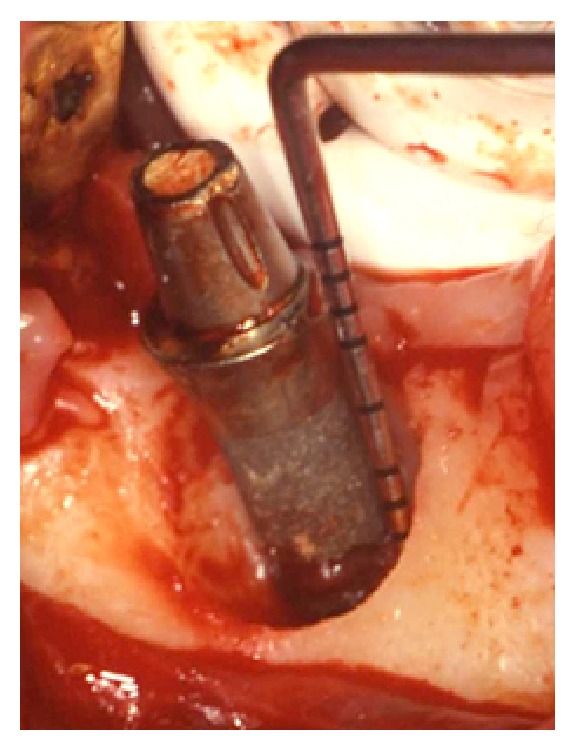
Clinical situation after the careful removal of the granulation tissue. The typical crater-like peri-implantitis bone defect was present circumferentially around the implant.

**Figure 5:**
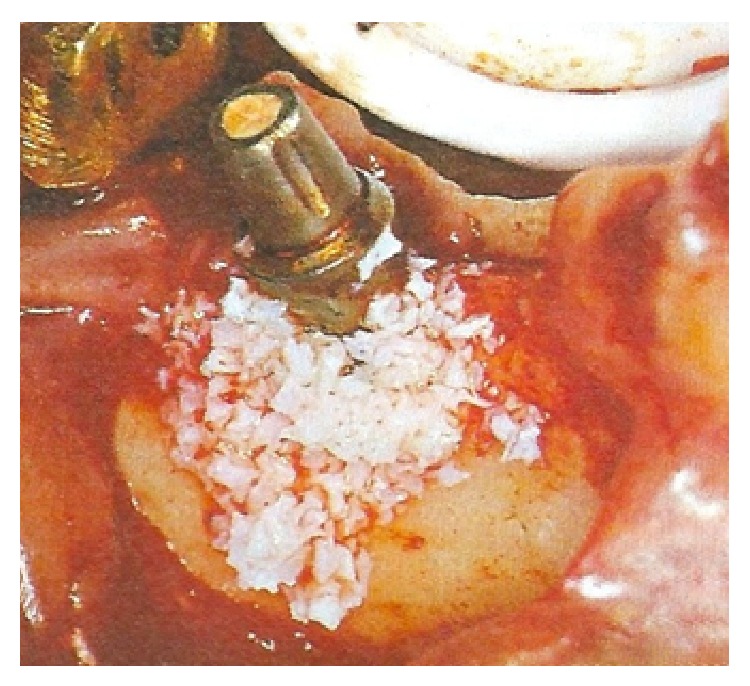
The bone defect was packed with heterologous DBBM graft.

**Figure 6:**
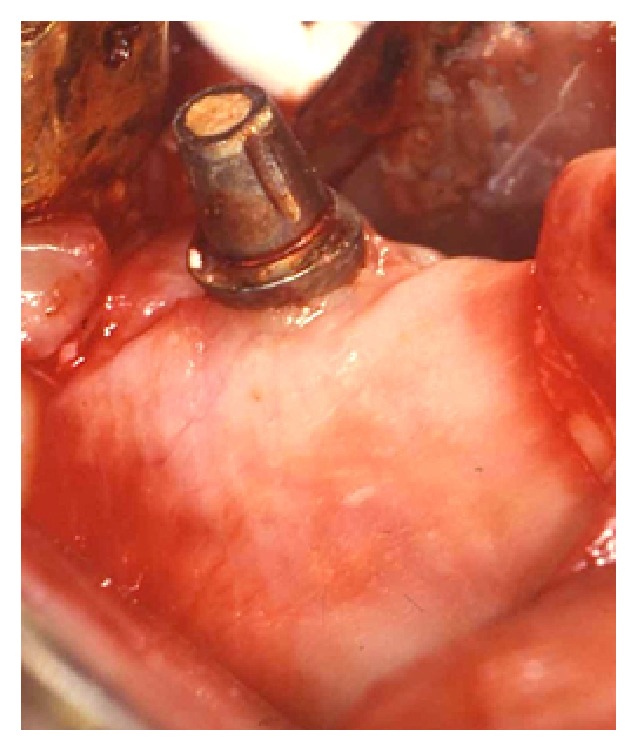
A bioabsorbable collagen membrane was placed in order to create a secluded space over the DBBM graft.

**Figure 7:**
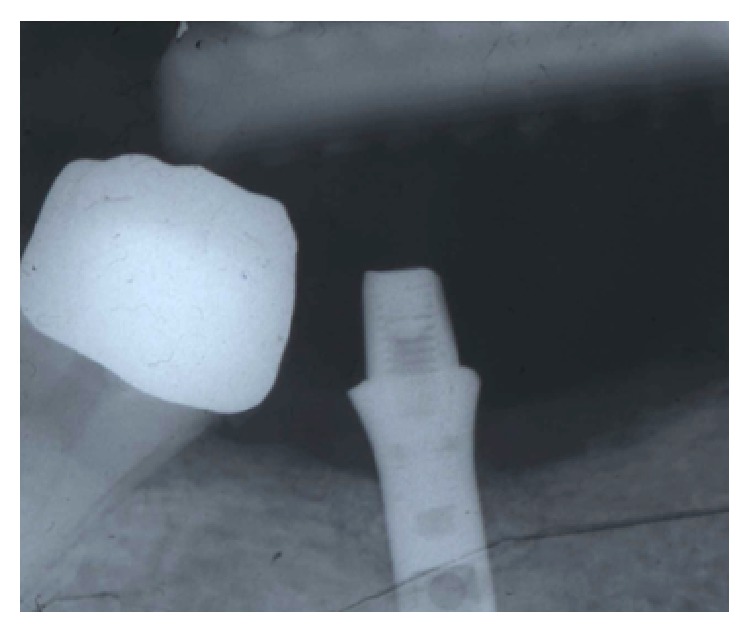
Postoperative intraoral radiography. The graft filled into the bone defect was observable in close contact with the implant surfaces. During the healing period the implant was left unloaded.

Once peri-implantitis was diagnosed, a nonsurgical periodontal supportive therapy was firstly performed to reduce the inflammation, followed by the surgical treatment of the defect. The latter consisted in the elevation of a full-thickness flap to have access to the bone defect and to mechanically remove the granulation inflammatory tissue around the implant (Figures 3 and 4). The mechanical decontamination was performed with Teflon curettes to minimise possible damages to the fixture surface. Concerning the chemical treatment, a 50 mg/mL tetracycline solution was topically applied with cotton pledged for 2 minutes on the exposed threads and was then rinsed off with physiological saline sterile irrigation. The bone defect was then grafted with deproteinized bovine bone mineral (DBBM) (Bio-Oss, Geistlich Pharma AG, Wolhusen, Switzerland) (Figure 5) and a resorbable collagen membrane (Bio-Gide, Geistlich Pharma AG, Wolhusen, Switzerland) was placed to protect the graft from the surrounding soft tissue cells penetration (Figure 6). Nylon 5/0 single stitches were applied to permit a primary intention wound healing. The implant remained without the prosthetic crown during the 6-month healing period. The healing abutment was not placed because of the high neck design of the transmucosal implant. Antibiotic therapy consisting in amoxicillin 500 mg three times a day was administrated during the following 7 days, associated with chlorhexidine 0,2% mouth rinses starting from the day after the surgery and extended to the next two weeks, in order to prevent postsurgical secondary infections. The sutures were removed 12 days after the surgery. Neither swelling nor pain occurred during the healing period. After 6 months the crown was replaced and an intraoral radiography was taken, showing increased radiopacity and complete filling of the defect with newly formed mineralized bone around the implant (Figure 7). The PD measurement was 1 mm and neither BOP nor mobility of the implant was recorded.

**Figure 8:**
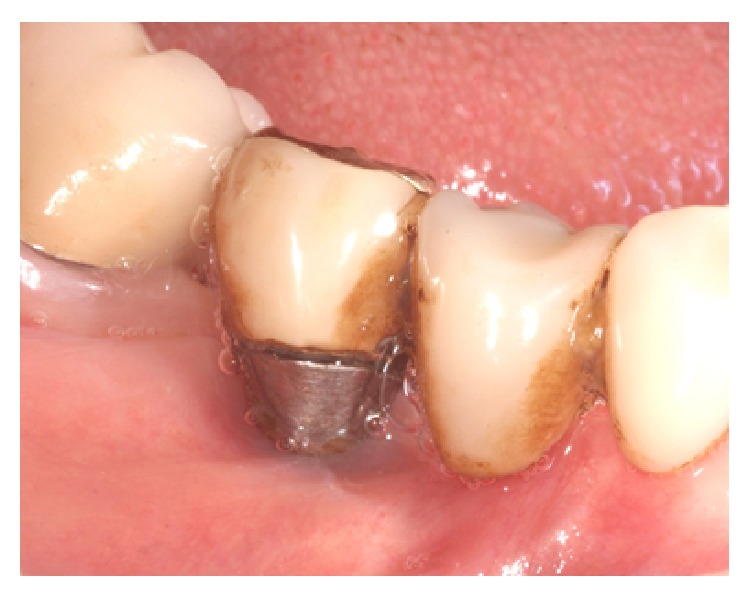
Clinical situation during the follow-up recall. Peri-implant soft tissues appeared healthy, with no sign of inflammation and suppuration.

**Figure 9:**
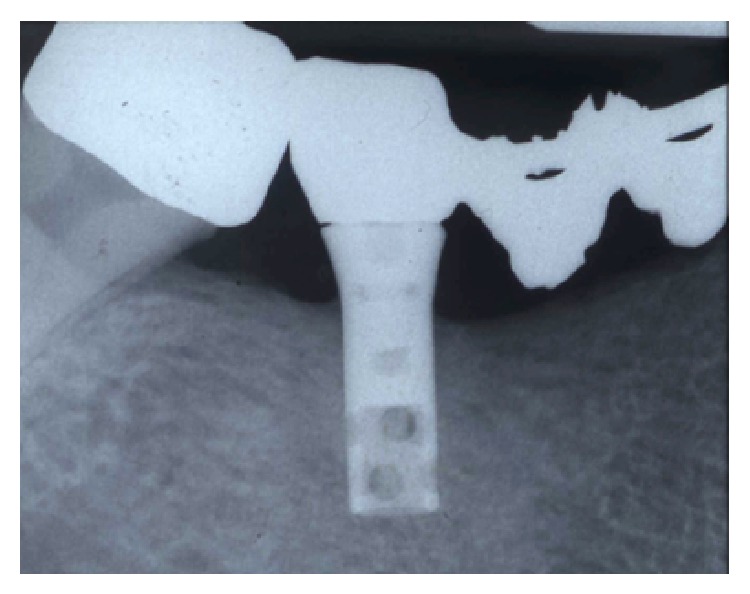
17-year follow-up intraoral radiography. The peri-implant marginal bone levels appeared radiographically stable, without any sign of bone resorption mesially and distally to the implant.

The patient underwent regular professional oral hygiene procedures every 6 months and a recall visit was conducted at least once a year. During the follow-up visit after 17 years, the implant was still supporting a fixed prosthetic crown. The peri-implant soft tissues presented no clinical signs of inflammation and BOP, and a physiological PD of 1.5 mm was measured (Figure 8). The mesial and distal levels of the peri-implant marginal bone were radiographically stable as demonstrated by the follow-up periapical X-rays (Figure 9).

## 3. Discussion

Several surgical procedures for the treatment of peri-implantitis could be found in literature. Nonsurgical therapy might be effective in the treatment of peri-implant mucositis. Furthermore, the adjunctive use of antimicrobial mouth rinses may enhance the outcome of the mechanical debridement in case of peri-mucositis. In peri-implantitis lesions however, nonsurgical therapy was found to be less effective. For this reason, surgical therapy and adjunctive local or systemic antibiotics may cooperate in the reduction of BOP and PD parameters [17]. Attempts have been made to determine the optimal conservative, regenerative, or resective surgical protocol to achieve the complete resolution of peri-implantitis, including the regeneration of lost tissues and the hypothetical reestablishment of osseointegration along previously contaminated implant surfaces, as described in the present case report. In these terms, bone defect configuration seems to play a key role concerning the clinical outcome following a surgical regenerative approach of peri-implantitis lesions, as suggested by Schwarz et al. [12]. Defects characterized by a deep vertical component with the buccal and lingual bone plates preserved have a more predictable outcome following regenerative therapy, as confirmed by the present case.

In this report, topical application of tetracycline solution for implant surface decontamination has proven to be an effective treatment option when coupled with bone regenerative procedures, as demonstrated by the long-term follow-up. Tetracyclines are broad-spectrum agents, exhibiting activity against a wide range of gram-positive and gram-negative bacteria, inhibiting protein synthesis by preventing the attachment of aminoacyl-tRNA to the ribosomal acceptor (A) site. The favourable antimicrobial properties of these agents and the absence of major adverse side effects have led to their extensive use in the therapy of human infections [18]. On the other hand, it was demonstrated that the development of resistance mechanisms may reduce the effectiveness of the molecule [19]. Even if tetracyclines in dentistry can be administrated systemically, recent trials indicated that locally delivered antimicrobials might enhance the effects of periodontal surgical therapy and reduce the signs of peri-implantitis [20]. The topical use in previously infected natural pockets has to be preferred, in order to reduce the probability of exogenous reinfection during the healing period [21]. Tetracyclines are released at effective concentrations over 7 days, and significant reductions in anaerobic pathogens up to 60% could be sustained for 6 months posttreatment [22]. Tetracycline fibers were originally applied topically in the treatment of periodontal diseases: affected sites had fibers placed to the depth of periodontal pockets, maintained in place by superficial applying of cyanoacrylate adhesive. Subsequently, the local use of tetracycline showed positive effects on clinical and microbiological parameters in the treatment of peri-implantitis [23]. Actually, this class of antibiotic showed improved clinical results when involved in the treatment of peri-implantitis in conjunction with bone grafts filled into the defect [24].

The choice of the graft material deserves some words to be spent. A wide selection has been used over the years for the treatment of peri-implantitis. The majority of peri-implantitis cases found in literature and treated with graft materials alone were located in the mandible. Although some failures were reported, most of the treatments generally resulted in improved clinical results. The studies by Behneke et al. included multiple defects approached by means of autogenous bone grafts with observation intervals extending for some cases up to 3 years [13, 25, 26]. Notable reductions of PD coupled with significant radiographic bone fill were reported. Out of the 25 consecutive lesions treated, failure and graft removal was reported only for two lesions. Other four lesions showed flap dehiscences within 2-3 weeks after grafting, which healed following chlorhexidine rinses or osteoplasty [13]. Guided bone regeneration may constitute a predictable way to treat osseous peri-implant defects improving soft tissues condition as well. Most of the studies concerning the treatment of peri-implantitis comprehended those in which a combination of grafts and barrier membranes was used. The submerged approach was used in half of these studies to allow for undisturbed healing and to reduce the risk of infection. From the results it was evident that the submerged approach was not always successful in practice, as the most common complication was the membrane exposure. A comparative study published by Khoury and Buchmann evaluated the use of autogenous bone grafts alone or associated with both bioabsorbable and nonresorbable membranes in a submerged approach. The results showed no significant differences between the groups in terms of treatment outcome after 3 years; however complications quite often occurred when membranes were used [14]. A study by Schwarz et al. evaluated the healing of intrabony peri-implant defects following application of a nanocrystalline hydroxyapatite paste or DBBM in combination with resorbable collagen membranes. Clinical parameters were recorded at baseline and after 6 months. Results showed that both treatments led to improved clinical conditions [27]. Recently, a study by Roos-Jansåker et al. compared two surgical techniques using a bone substitute with or without the application of bioabsorbable membranes adopting a nonsubmerged approach. According to Khoury et al. no significant differences were observed between either group [14, 15]. It could be concluded that the placement of membranes in addition to bone grafting does not provide any adjunctive effect. Considering the present case report, at the time of intervention much of this knowledge was still unknown; however it could be assumed that the presence of a bioabsorbable membrane may have promoted an undisturbed graft remodelling, permitting a new bone formation due to the creation of a secluded space over the graft against the ingrowth of soft tissue cells surrounding the defect. Moreover, differently from nonresorbable membranes, the bioabsorbable collagen membrane used in the present clinical case may have decreased the risk of membrane exposure, allowing an uneventful healing as demonstrated by the 6-month recall visit.

Peri-implant disease treatment is still a controversial topic, as several authors provided different therapeutic solutions [28]. A correct diagnosis represents the first and most important step in order to schedule an adequate treatment and its relevance is univocally acknowledged. In 2004 Lang et al. published important guidelines based on a diagnostic-therapeutic algorithm [29]. In presence of a severe level of the disease, in addition to the implant surface decontamination and antibiotic therapy, a surgical approach should be performed. When regenerative treatment is chosen, the use of a barrier membrane technique, even in combination with bone substitutes like DBBM, is recommended.

## 4. Conclusion

The results obtained in the present report stressed the importance of a combined antibiotic and mechanical decontamination associated with a regenerative procedure in the treatment of seriously compromised implants. This treatment led to positive effects on the clinical and radiological parameters over a long-term follow-up timespan.
